# Influence of Emulsifying Salts on the Growth of *Bacillus thuringiensis* CFBP 3476 and *Clostridium perfringens* ATCC 13124 in Processed Cheese

**DOI:** 10.3390/foods11203217

**Published:** 2022-10-14

**Authors:** Andressa Fusieger, Raiane Rodrigues da Silva, Valéria Quintana Cavicchioli, Rafaela da Silva Rodrigues, Jaqueline Aparecida Honorato, Sidney Rodrigues de Jesus Silva, Mariana Lage Pena, Cinzia Caggia, Luís Augusto Nero, Antonio Fernandes de Carvalho

**Affiliations:** 1 InovaLeite-Laboratório de Pesquisa em Leites e Derivados, Departamento de Tecnologia de Alimentos, Universidade Federal de Viçosa, Viçosa 36570 900, MG, Brazil; 2 InsPOA-Laboratório de Inspeção de Produtos de Origem Animal, Departamento de Veterinária, Universidade Federal de Viçosa, Viçosa 36570 900, MG, Brazil; 3 Di3A-Dipartimento di Agricoltura, Alimentazione e Ambiente, Università degli Studi di Catania, 95123 Catania, CT, Italy; 4 Centro de Pesquisa em Alimentos, Escola de Veterinária e Zootecnia, Universidade Federal de Goiás, Goiânia 74690 900, GO, Brazil

**Keywords:** dairy, food safety, melting salts, microbiological stability, polyphosphate, shelf life, spore-forming bacteria

## Abstract

Processed cheese is a dairy product with multiple end-use applications, where emulsifying salts play a fundamental role in physicochemical changes during production. Moreover, some of these salts may be a strategy to control spoilage and pathogenic microorganisms, contributing to safety and shelf life extension. This study aimed to evaluate the in vitro inhibitory activity of two emulsifying salts (ESSP = short polyP and BSLP = long polyP) against *Bacillus thuringiensis* CFBP 3476 and *Clostridium perfringens* ATCC 13124, and to compare the in situ effects of two emulsifying salts treatments (T1 = 1.5% ESSP and T2 = 1.0% ESSP + 0.5% BSLP) in processed cheeses obtained by two different methods (laboratory- and pilot-scales), during 45-day storage at 6 °C. *C. perfringens* ATCC 13124 growth was not affected in vitro or in situ (*p* > 0.05), but both of the treatments reduced *B. thuringiensis* CFBP 4376 counts in the tested condition. Counts of the treatments with B. *thuringiensis* CFBP 3476 presented a higher and faster reduction in cheeses produced by the laboratory-scale method (1.6 log cfu/g) when compared to the pilot-scale method (1.8 log cfu/g) (*p* < 0.05). For the first time, the inhibitory effect of emulsifying salts in processed cheeses obtained by two different methods was confirmed, and changes promoted by laboratory-scale equipment influenced important interactions between the processed cheese matrix and emulsifying salts, resulting in *B. thuringiensis* CFBP 4376 growth reduction.

## 1. Introduction

Processed cheese is a relatively new category of dairy products, set up a little over 100 years ago independently in Europe and the United States. Driven by the need to increase the shelf life of natural cheese and with the possibility of recycling defective cheeses, processed cheeses have emerged with a distinct texture, flavor and/or functional properties. Through a technology able to standardize these properties, a versatile product was introduced to the market [1,2,3,4]. From a technological point of view, processed cheese is obtained by heating a mixture of cheeses, under partial vacuum conditions and constant stirring, in the presence of emulsifying salts which are able to chelate calcium, disrupting the casein structure and solubilizing it [1,5]. The process can be performed by using different equipment, in which various types of cookers with different designs and operating conditions can be employed. The equipment may differ mainly on the type of mixing or agitating systems, the type and mechanism of heating, and the mode of production, in batch or continuous, according to the industry needs [6,7]. The appropriate selection of the equipment and the type and quantity of emulsifying salts are among the most important variables to produce processed cheese with desired final properties [8,9].

During processed cheese production, monophosphates, polyphosphates (polyP) and citrates are emulsifying salts commonly used [5]. These salts consist of a monovalent cation, typically sodium, bound to a charged polyvalent anionic tail that acts as a calcium sequester, involved in the disruption of the calcium–phosphate-linked protein network present in natural cheese. Both functions have an effect in hydrating the caseins, allowing the interaction between water and fatty phases, thereby producing a homogeneous cheese emulsion [5,8].

The use of different emulsifying salts with different emulsification capabilities is a growing practice in the dairy industry [5,10,11]. Furthermore, based on the antimicrobial effect of some emulsifying salts, the addition of phosphates has recently been proposed as a strategy to control spoilage and pathogenic microorganisms, and ultimately, to reduce the sodium content and prolong the shelf life of processed cheese [5,12,13].

Most of the studies on emulsifying salts in processed cheese have been focused on mechanical, physicochemical, microstructure, and rheology properties, as well matrix stability, formation of casein fibrils and sensory traits [10,11,14,15,16,17]. However, the number of scientific studies on the antimicrobial potential of emulsifying salts is low and few studies have been published in the last five years [13,18,19]. Processed cheeses have been associated with certain microbiological safety concerns and despite the lower susceptibility to microbial spoilage, spore-forming bacteria, such as *Bacillus thuringiensis* and *Clostridium perfringens* have been associated with blowing and putrid odor development [20,21]. Although the spore-forming bacteria originated mainly from raw milk, the production environment can also act as a source of contamination, due to the ability of these microorganisms to survive cleaning and sanitation steps [22]. For this reason, the addition of emulsifying salts can be considered in many processed cheese productions as a control option.

Based on the above scenario, this is the first study aimed at comparing the inhibitory activity of emulsifying salts in processed cheese obtained by following different processes. In particular, the inhibitory activity of two emulsifying salts against *B. thuringiensis* CFBP 3476 and *C. perfringens* ATCC 13124 inoculated in processed cheese obtained on a laboratory-scale and a pilot-scale was explored.

## 2. Materials and Methods

### 2.1. Materials and Treatments

Two mixtures of emulsifying salts composed of polyphosphates (E452) and sodium phosphates (E339) were used in this study. These mixtures differ in the total phosphate content (P2O5), the average chain length, and pH (1% solution), as follows: (ESSP) emulsifying salt composed of short polyP, used as an emulsifier agent, 59.7 ± 1% P2O5, short-chain polyphosphate, pH 9.0 ± 0.3; (BSLP) bacteriostatic salt composed of long polyP, used as a bacteriostatic specialty, 69 ± 1% P2O5, long-chain polyphosphate, pH 6.0 ± 0.5.

To evaluate the inhibitory activity of emulsifying salts by in vitro and in situ approaches, two treatments were performed: Treatment 1 (T1) = 1.5% ESSP; and Treatment 2 (T2) = 1.0% ESSP + 0.5% BSLP.

The inhibitory activity of the emulsifying salts was tested against *Bacillus thuringiensis* CFBP 3476 and *Clostridium perfringens* ATCC 13124. The strains were stored at −80 °C in a culture medium and 20% (*v*/*v*) glycerol. For strains cultivation, incubation was in a brain heart infusion broth (BHI; Kasvi, São José dos Pinhais, Brazil) for 22 ± 2 h at 30 °C was used for *B. thuringiensis* CFBP 3476 and trypticase soy broth (TSB; Kasvi) for 22 ± 2 h at 37 °C under anaerobiosis was used for *C. perfringens* ATCC 13124. Before use, the strains were transferred into a broth medium, incubated and checked for purity in BHI or TSA agar (Kasvi) after the incubation time. Then, a single colony was transferred to the appropriate broth medium, incubated, and diluted to turbidity similar to a 0.5 standard on the MacFarland scale, equivalent to 1.5 × 10^8^ cfu/mL.

For the assessment of in vitro inhibitory activity, these cultures were diluted on a decimal scale to approximately 1.5 × 10^6^ cfu/mL. For the assessment of in situ inhibitory activity, the fresh cultures were centrifuged (Heraeus Megafuge 8R, Thermo Fisher Scientific, Waltham, MA, USA) at 3260× *g* for 15 min at 7 °C. After discarding the supernatant, the pellets were suspended in NaCl solution 0.85% (*w*/*v*) until turbidity similar to a 0.5 standard on the MacFarland scale, corresponding to approximately 1.5 × 10^8^ cfu/mL. Cultures of *B. thuringiensis* CFBP 3476 and *C. perfringens* ATCC 13124 were separately used as primary inoculum in the processed cheese.

### 2.2. In Vitro Inhibitory Activity

To detect the in vitro inhibitory activity, a streak assay protocol was performed on nutrient agar (NT; Kasvi) [23]. Briefly, NT agar was prepared according to the manufacturer’s instructions (Kasvi), autoclaved (121 °C for 15 min), and cooled to 45 °C, after which the emulsifying salts were added. To simulate the processed cheese processing, agar solutions were homogenized, heated for 5 min at 90 °C in a water bath (MA156/6, Marconi, Piracicaba, Brazil), and poured into Petri dishes. *B. thuringiensis* CFBP 3476 and *C. perfringens* ATCC 13124 at approximately 1.5 × 10^6^ cfu/mL were streaked onto the plates and incubated under the appropriate conditions for each strain for 48 h. Sterile water was used as a negative control and agar plates without any addition of emulsifying salts were considered as a control treatment for bacterial growth. After incubation, the absence of bacterial growth was indicative of the inhibitory activity of the emulsifying salt treatments. This assay was conducted in duplicate and with three independent repetitions.

### 2.3. In Situ Inhibitory Activity

#### 2.3.1. Production of Processed Cheese

To produce processed cheese, the following ingredients and concentrations were used: Mozzarella cheese aged for 2 weeks, 49% (*w*/*w*); butter, 17% (*w*/*w*); sterile mineral water, 39.5% (*w*/*w*), and emulsifying salts according to treatments T1 and T2. For each treatment, the production was carried out under aseptic conditions and the processing was performed using two different methods: (I) laboratory-scale, using a Thermomix TM-5 (Vorwerk & Co. Thermomix; GmbH, Wuppertal, Germany) with a 2 kg capacity, and (II) pilot-scale, using Stephan Geiger homogenizer-grinder GUMSK 12E NR12 (Geiger Indústria de Máquinas Ltd.a., Pinhais, PR, Brazil) with a 7 kg capacity. The ingredients were blended with subsequent processing by heating for 6 min until reaching a 90 °C melting temperature, and shearing with a constant increase in rotation until 1100 rpm for the laboratory-scale method, and until 1500 rpm for the pilot-scale method; the mixing speed was performed for 5 min at 90 °C.

**Figure 1:**
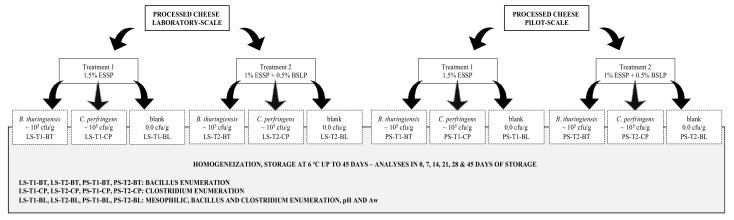
Flow chart of procedures employed in the production of processed cheese samples and the application of treatments to assess the inhibitory activity of emulsifying salts.

Then, four 200 g portions of each processed cheese treatment were split into sterile bags (polyethylene bags; height 178 mm, width 76 mm, and thickness 0.07 mm; Kasvi) and cooled to 12 ± 2 °C. Two portions of each treatment were separately inoculated with each target strain (described above) at a final concentration of 1.5 × 10^5^ cfu/g and homogenized for 5 min (Bagmixer 400 VW; Interscience, Paris, France). The inoculated processed cheese was equally divided into nine sterile bags (25 g) and stored at 6 ± 2 °C for 45 days. In the next step, one portion of each processed cheese treatment was not inoculated and considered as the negative control samples (blank), and another portion was also not inoculated and used to monitor the pH and Aw during the storage time; each of them was divided equally into six sterile bags (25 g) and kept at the same storage conditions. Two independent repetitions of each processed cheese treatment were performed. For each repetition, different batches of ingredients (cheese, butter, sterile mineral water, and emulsifying salts) were used. The flow chart of the experimental design is reported in Figure 1.

#### 2.3.2. Microbiological Analyses

Processed cheese samples were subjected to microbiological analyses during the 45-day storage at 6 ± 2 °C (at day 0, day 7, day 14, day 21, day 28, and day 45). In detail, a 25 g of sample was added to 225 mL of citrate solution 2% (*w*/*v*), homogenized, and 10-fold-diluted in NaCl solution 0.85% (*w*/*v*). For samples inoculated with *B. thuringiensis* CFBP 3476, aliquots of 100 µL of selected dilutions were plated onto mannitol egg yolk polymyxin (MYP; K25-1343, Kasvi) agar supplemented with egg yolk and polymyxin B (K25-6021, Kasvi) and incubated at 30 ± 1 °C for 18–24 h [24,25]. For samples inoculated with *C. perfringens* ATCC 13124, aliquots of 1 mL were pour-plated on tryptose sulfite cycloserine (TSC; K25-1029, Kasvi) agar supplemented with *Clostridium perfringens* selective supplement (K25-6020, Kasvi) and incubated under anaerobic conditions at 37 ± 1 °C for 24 h [26]. Control samples (blank samples) were analyzed following the same protocols described above and for total viable counting in plate count agar (PCA; Kasvi) incubated at 30 ± 1 °C for 72 h [27]. The results were expressed as colony-forming units per gram and counts were converted into Log10 (Log10 cfu/g). These analyses were conducted in triplicate and over two independent repetitions of each processed cheese treatment.

#### 2.3.3. Water Activity and pH

The water activity (Aw) of blank processed cheese samples was measured using a water activity meter (AquaLab 3TE; Decagon Devices Inc., Pullman, WA, USA). The sample cup was filled to half its depth and placed in the sample chamber and the Aw was measured using the standard procedure. The pH of blank processed cheese samples was measured using a pH meter (Hanna Instruments Ltd., Leighton Buzzard, UK). These analyses were conducted in duplicate and over two independent repetitions of each processed cheese treatment.

### 2.4. Statistical Analysis

Statistical analyses were performed using SAS^®^ Studio software (Release: 3.8; Enterprise Edition; SAS Institute, Cary, NC, USA). Data were checked for normality of residuals and homogeneity of variances using the Kolmogorov–Smirnov test and Bartlett’s test, respectively, using the GLM and CAPABILITY procedures. Then, data were compared by ANOVA and Tukey tests (*p* < 0.05), using the GLM procedure. The R software, version 4.0.2 (The R Foundation, Boston, MA, USA), and the RStudio, version 1.3.959 (Integrated Development for R. RStudio, Boston, MA, USA), were used for graphical presentation with the ggplot2 package [28].

## 3. Results

### 3.1. In Vitro Inhibitory Activity

Results of the in vitro inhibitory activity, for both treatments T1 (1.5% ESSP) and T2 (1.0% ESSP + 0.5% BSLP), showed that *B. thuringiensis* CFBP 4376 growth was inhibited, whereas *C. perfringens* ATCC 13124 was not inhibited.

### 3.2. In Situ Inhibitory Activity

Plate counts for the processed cheese blank samples (without inoculation) did not present viable and cultivable cells during storage time. For the inoculated processed cheese samples, different profiles were found and the results are reported below.

**Figure 2:**
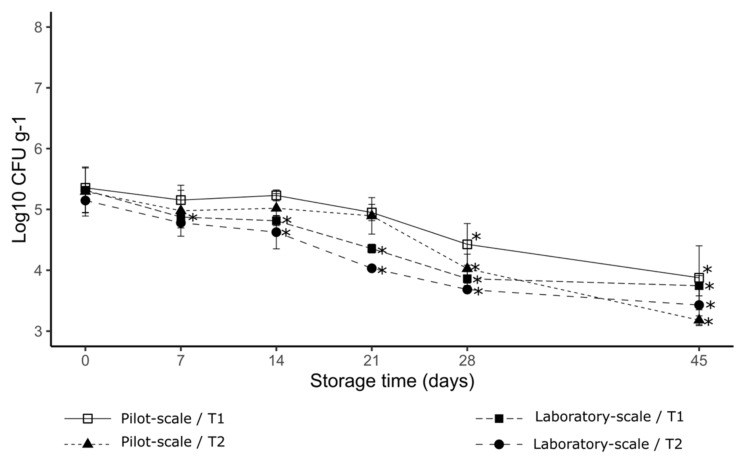
The effect of emulsifying salts on the inactivation of *B. thuringiensis* CFBP 4376, with different processing methods over storage time. (*) indicates a significant difference with the zero time of storage, according to Tukey’s test (*p* < 0.05). Error bars show standard deviations of the mean.

All treatments showed a significant effect on the inactivation of *B. thuringiensis* CFBP 4376 in both production methods (laboratory and pilot-scale). In comparison with time zero, there was a significant difference (*p* < 0.05) from day 28 and 45 of storage in both production methods. In addition, for the laboratory-scale method a significant difference was observed after day 7 of storage for T1, and after day 14 of storage for T2 (Figure 2). Higher levels of inhibition were recorded for T2 after 45 days of storage, being 3.43 Log10 CFU/g for the laboratory-scale and 3.18 Log10 CFU/g for the pilot-scale.

**Figure 3:**
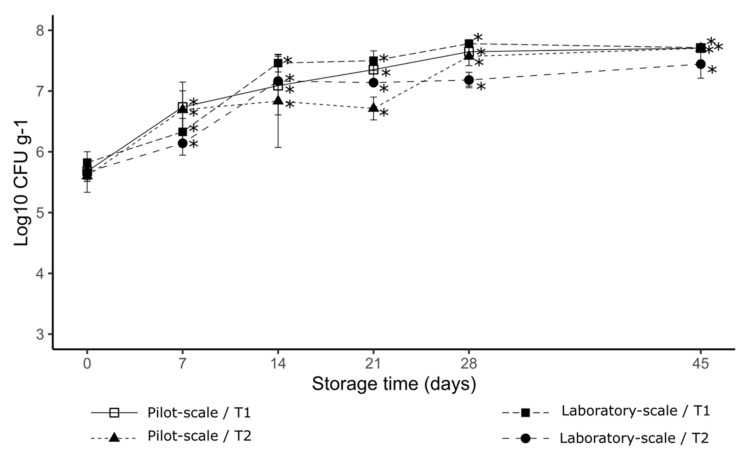
The effect of the emulsifying salts on *C. perfringens* ATCC 13124 development, with different processing methods over storage time. (*) indicates a significant difference with the zero time of storage, according to Tukey’s test (*p* < 0.05). Error bars show standard deviations of the mean.

According to Figure 3, none of the treatments showed an inhibitory effect on the multiplication of *C. perfringens* ATCC 13124. From the day 7 storage samples, the values of Log10 CFU/g increased and differed significantly from time zero (*p* < 0.05) and for both production methods. Between day 28 and day 45, a stationary phase was observed for all treatments.

### 3.3. Water Activity and pH

Blank samples produced at the pilot-scale showed significantly different pH values over time of storage for T2 (Table 1). For T1, only at day 21 of storage did the pH value not differ from time zero (*p* > 0.05). However, the pH of the blank samples was stable when produced at the laboratory-scale, since, in both treatments, there was no significant variation during storage (*p* > 0.05) compared to time zero.

According to the analysis of variance of the results obtained for water activity (Aw, Table 2), there was no statistical difference between the analyzed treatments (*p* > 0.05).

## 4. Discussion

The BSLP shows both bacteriostatic and fungistatic effects and prevents the outgrowth of aerobic and anaerobic spores, although the dosage rate depends on the microbial species and the matrix (according to the manufacturer’s information). As BSLP is a long-chain polyP, its use is recommended simultaneously with other emulsifying salts, such as ESSP, mainly in spreadable and sliceable processed cheese production. In the present study, a standard formulation of ESSP, commonly applied in the dairy industry, and an additional formulation, with partial replacement by BSLP were investigated. While *C. perfringens* ATCC 13124 growth was not affected by any of the tested treatments, neither in vitro nor in situ, both treatments with PolyP emulsifying salts were able to control *B. thuringiensis* CFBP 4376 outgrowth, in both in vitro and in situ.

Buňková et al. [29] performed an in vitro study on the antimicrobial effects of three commercially available emulsifying salts on reference microorganisms and bacteria isolated from long-stored processed cheeses. The results of the present study agree with those reported by Buňková et al. [29], which described the positive inhibition of 0.5% emulsifying salts with 69.0% P2O5 treatment (the same P2O5 level as reported here for BSLP) against *B. cereus* CCM 3953, *Bacillus subtilis* CCM 2216, *Bacillus brevis* SPSM 4101, *Bacillus sphaericus* CCM 1615, *Bacillus stearothermophilus* SPSM 4103, *Bacillus* sp. NTS 01, NTS 02, NTS 03, NTS 05, and NTS 06. However, the same study reported that 0.5% emulsifying salts with a 59.7% P2O5 treatment (the same P2O5 level as reported here for ESSP) were not able to inhibit the growth of *B. subtilis* CCM 2216, *B. sphaericus* CCM 1615, *Bacillus* sp. NTS 01, NTS 03, NTS 05, and NTS 06. Lorencová et al. [30] carried out a study on the antibacterial effect of seven emulsifying salts at different chain lengths and different phosphate contents; the authors pointed out that the inhibitory effect can be affected by intrinsic factors, such as the number of phosphorus atoms and the acid–basic properties of emulsifying salts in aqueous solutions.

For processed cheese samples, the inactivation of *B. thuringiensis* CFBP 4376 growth by the emulsifying salt treatments was verified. Although the emulsifying salts had an effect on the inactivation of *B. thuringiensis* CFBP 4376 growth, a bacterial growth reduction was reached more rapidly along the storage time when the laboratory-scale method was applied; the means of T1 and T2 resulting in a 1.64 log unit reduction for laboratory-scale and in a 1.8 log unit reduction for pilot-scale samples at the end of storage time. The main differences between the two production methods were that on the laboratory-scale, no steam injection was performed and an 1100 rpm shearing was applied, while on the pilot-scale an indirect steam injection and a 1500 rpm shearing were performed. According to Guinee [8], a higher agitation speed during processing significantly increases the firmness and elasticity modulus of processed cheese and significantly reduces the level of flow and the fluidity of the melted product. In addition, the general bacteriostatic effect of phosphates may reflect interactions with bacterial proteins and the sequestration of calcium, which generally serves as an important cellular cation and cofactor for some microbial enzymes.

Species belonging to the genus *Bacillus* are commonly isolated from processed cheese [22,31] and a major focus is placed on the role of *B. cereus* in foodborne disease outbreaks. Furthermore, *B. thuringiensis* belongs to the phylogenetic group of *B. cereus* and it is biochemically identical to the *B. cereus* species [32]. Therefore, the occurrence of foodborne outbreaks of *B. thuringiensis* may be underestimated because routine microbial diagnostics of *B. cereus* and *B. thuringiensis* are not differentiated [33]. Therefore, the effects of the emulsifying salts observed here may be a useful strategy to control *Bacillus* spp. in processed cheese.

In addition, the *B. thuringiensis* CFBP 4376 growth inactivation can be explained by the mechanisms already described for *B. cereus*. According to Maier et al. [34], the antibacterial activity of polyphosphates with a high level of P2O5, such as BSLP, against *B. cereus* is related to the interaction of long-chain polyP in the exponential growth phase, affecting the cell morphology manifested by cell lysis and the inability of septum formation during division. Briefly, the authors proposed that polyP might influence the ubiquitous bacterial cell division protein FtsZ, whose GTPase activity is known to be strictly dependent on divalent metal ions.

For processed cheese samples inoculated with *C. perfringens* ATCC 13124, the bacterial growth was not inhibited under the tested conditions. Loessner et al. [19] evaluated the effect of long-chain polyP formulations on the growth of *Clostridium tyrobutyricum* ATCC 25755 in processed cheese spreads. Although the inoculation of spores was performed, the samples were processed with the addition of an emulsifying salt with 59.7% P2O5, and only at the end of the process was an aqueous solution of emulsifying salts with higher levels of P2O5 added to the samples. This is not a common procedure followed at an industrial scale. However, no viable cell was detected after 8 days of incubation by 0.5% emulsifying salts with higher levels of P2O5, and total inhibition was obtained until 50 days of storage in samples treated with 1.0% emulsifying salts with higher levels of P2O5 [19]. On the other hand, Akhtar et al. [35] performed a study to evaluate the effects of various polyPs (sodium polyP, tetrasodium pyrophosphate, sodium tripolyphosphate, and sodium acid pyrophosphate) on growth, sporulation, and spore germination of *C. perfringens*, and germination and outgrowth of *C. perfringens* spores. The authors pointed out that surprisingly, all 19 *C. perfringens* vegetative cells required higher polyP concentrations (1.0–1.4%) than those previously reported for other bacteria, such as *Staphylococcus aureus*, *B. cereus*, *Listeria monocytogenes*, *C. tyrobutyricum*, *C. pasteuranium*, and *C. butyricum*, where the minimum inhibitory concentration, independently of the type of polyP, an in vitro assay was at most 0.5%. According to Akhtar et al. [35], the high polyP resistance of *C. perfringens*, compared to other bacteria, could be attributed to the higher synthesis of phosphatases producing higher rates of polyP hydrolysis, and/or to differences in the cell wall structure.

In general, it is difficult to determine the polyP chain length and commonly only approximate estimations can be found in the literature. Therefore, polyP is characterized by the P2O5 content, which may reflect the average size of phosphate chains in a mixture, and the higher the P2O5 content, the greater the size of the chain; its concentration is given as a percentage rather than a molar [19,36]. BSLP is composed of 69.0 ± 1% P2O5, while ESSP of 59.7 ± 1% P2O5; consequently, the chain size of the BSLP is larger. The inhibitory effect of polyP is related to the length of the chain (condensation level), and polyP with a lower amount of phosphorus is generally less effective in the suppression of undesirable microorganism growth than long-chain phosphates [12,30]. Long-chain polyP are characterized by a high affinity for divalent metal ions (Ca^2+^ and Mg^2+^), essential for the integrity of Gram-positive bacteria cell walls [5,37]. According to the above-mentioned results, the BSLP treatment was more effective against *C. perfringens* ATCC 13124 until 45 days of storage, even if no differences were observed for *B. thuringiensis* CFBP 4376.

Overall, the effect of polyP is mainly related to changes in environmental conditions where they induce changes in the pH and ionic strength of the solution, modifying protein configuration, solubility, and to the last extent, emulsifying salts bind Ca^2+^ [5,12]. PolyP are used to shift the pH of the cheese upwards, typically from 5.0–5.5 of natural cheese to 5.6–6.0 of pasteurized processed cheese, and the stabilization that occurs may be due to their high buffering capacity [1]. During processed cheese production, the pH change contributes to an enhanced dissociation and calcium-sequestering ability of the emulsifying salts, and an increased negative charge of the para-caseinate [1]. Thus, at a higher pH, more complete dissociation of the phosphate molecules provides better ion exchange.

Different emulsifying salts influence the final properties of processed cheeses due to their ability to exchange calcium ions for sodium ions and to stabilize pH values (buffering capacity) [38]. The ion exchange ability increases with the length of the polyphosphate chain [38,39]. In this way, processed cheese with the addition of short-chain emulsifying salts has a higher pH compared to the addition of long-chain emulsifying salts, as was the case for T1 and T2, respectively.

Regarding the Aw, one of the basic conditions for microbial growth, Awad et al. [40] evaluated the influence of different emulsifying salt mixtures on the Aw of block-processed cheese during storage at different temperatures for up to three months. The authors stated that neither the different combinations of emulsifying salts nor storage had a significant effect on the Aw of samples. In the present study, the detected Aw values are in accordance with those reported by Kim et al. [41] who, evaluating 800 processed cheese samples, found Aw values ranging from 0.8 to 1.0. In particular, the Aw values reported for cream cheese and cheese portions (0.97 ± 0.01 and 0.97 ± 0.02, respectively) were very close to the results reported in the present study (0.98 ± 0.004).

Finally, considering that the microbiological stability of processed cheese, as a low acidic complex food matrix, remains a challenge, the application of the hurdle theory represents the best strategy. In this scenario, the addition of polyP together with the microbiological quality of milk and other ingredients used in the manufacture of processed cheese becomes necessary. With the present study, for the first time, the inhibitory effect of emulsifying salts against *B. thuringiensis* in processed cheese obtained by two different methods was confirmed. Further studies on the use of different emulsifying salts are required to better establish microbiologically and technologically available formulations useful for the cheese industry, nevertheless, these preliminary findings must be considered as the first steps toward the application of multifunctional ingredients in processed cheeses.

## Figures and Tables

**Table 1:** The pH results were obtained according to the methods, treatments and storage time applied to blank processed cheese samples.

Storage Time (Days)	Pilot-Scale		Laboratory-Scale	
	T1	T2	T1	T2
0	5.88 ± 0.04 ab	5.77 ± 0.01 a	5.74 ± 0.01 a	5.52 ± 0.04 a
7	5.72 ± 0.04 d	5.49 ± 0.06 e	5.76 ± 0.12 a	5.52 ± 0.06 a
14	5.83 ± 0.07 bc	5.53 ± 0.01 de	5.87 ± 0.11 a	5.60 ± 0.04 a
21	5.92 ± 0.03 a	5.67 ± 0.04 b	5.75 ± 0.03 a	5.52 ± 0.04 a
28	5.78 ± 0.01 cd	5.59 ± 0.04 cd	5.80 ± 0.05 a	5.54 ± 0.07 a
45	5.85 ± 0.01 bc	5.63 ± 0.01 bc	5.78 ± 0.06 a	5.52 ± 0.03 a

Data are presented as mean ± standard deviation. Different letters within the column indicate a significant difference between storage times in each treatment, according to Tukey’s test (*p* < 0.05).

**Table 2:** The Aw results were obtained according to the methods, treatments and storage time applied to blank processed cheese samples.

Storage Time (Days)	Pilot-Scale		Laboratory-Scale	
	T1	T2	T1	T2
0	0.983 ± 0.001	0.982 ± 0.001	0.985 ± 0.002	0.975 ± 0.010
7	0.985 ± 0.002	0.986 ± 0.001	0.983 ± 0.001	0.984 ± 0.001
14	0.991 ± 0.005	0.987 ± 0.005	0.990 ± 0.002	0.991 ± 0.007
21	0.983 ± 0.001	0.985 ± 0.002	0.979 ± 0.009	0.980 ± 0.012
28	0.984 ± 0.000	0.984 ± 0.002	0.986 ± 0.012	0.987 ± 0.010
45	0.984 ± 0.001	0.984 ± 0.001	0.983 ± 0.004	0.980 ± 0.004

Data are presented as mean ± standard deviation. No significant difference was observed according to Tukey’s test (*p* > 0.05).

## Data Availability

All related data and methods are presented in this paper. Additional inquiries should be addressed to the corresponding author.

## References

[1] Fox P.F., Guinee T.P., Cogan T.M., McSweeney P.L.H. (2017). Processed Cheese and Substitute/Imitation Cheese Products. Fundamentals of Cheese Science.

[2] Ramel P.R., Marangoni A.G. (2018). Processed Cheese as a Polymer Matrix Composite: A Particle Toolkit for the Replacement of Milk Fat with Canola Oil in Processed Cheese. Food Res. Int..

[3] Farahat E.S.A., Mohamed A.G., El-Loly M.M., Gafour W.A.M.S. (2021). Innovative Vegetables-Processed Cheese: I. Physicochemical, Rheological and Sensory Characteristics. Food Biosci..

[4] Vollmer A.H., Kieferle I., Youssef N.N., Kulozik U. (2021). Mechanisms of Structure Formation Underlying the Creaming Reaction in a Processed Cheese Model System as Revealed by Light and Transmission Electron Microscopy. J. Dairy Sci..

[5] Buňka F., Černíková M., Salek R.N., El-Bakry M., Mehta B.M. (2022). Chapter 6—Functionality of Salts Used in Processed Cheese Manufacture. Processed Cheese Science and Technology.

[6] Noronha N., O’Riordan E.D., O’Sullivan M. (2008). Influence of Processing Parameters on the Texture and Microstructure of Imitation Cheese. Eur. Food Res. Technol..

[7] McIntyre I., O’Sullivan M., O’Riordan D. (2017). Monitoring the Progression of Calcium and Protein Solubilisation as Affected by Calcium Chelators during Small-Scale Manufacture of Casein-Based Food Matrices. Food Chem..

[8] Guinee T.P., Carić M., Kaláb M., Fox P.F., McSweeney P.L.H., Cogan T.M., Guinee T.P. (2004). Pasteurized Processed Cheese and Substitute/Imitation Cheese Products. Cheese: Chemistry, Physics and Microbiology.

[9] Talbot-Walsh G., Kannar D., Selomulya C. (2018). A Review on Technological Parameters and Recent Advances in the Fortification of Processed Cheese. Trends Food Sci. Technol..

[10] Nogueira E.B., Costa-Lima B.R.C., Torres F., Regazone A.V., Melo L., Franco R.M., Cortez M.A.S. (2018). Effect of Potassium-Based Emulsifying Salts on the Sensory and Physicochemical Parameters of Low-Sodium Spreadable Processed Cheese. Int. J. Dairy Technol..

[11] Mozuraityte R., Berget I., Mahdalova M., Grønsberg A., Øye E.R., Greiff K. (2019). Sodium Reduction in Processed Cheese Spreads and the Effect on Physicochemical Properties. Int. Dairy J..

[12] Buňková L., Buňka F. (2017). Microflora of Processed Cheese and the Factors Affecting It. Crit. Rev. Food Sci. Nutr..

[13] Martinez-Rios V., Jørgensen M.Ø., Koukou I., Gkogka E., Dalgaard P. (2019). Growth and Growth Boundary Model with Terms for Melting Salts to Predict Growth Responses of *Listeria monocytogenes* in Spreadable Processed Cheese. Food Microbiol..

[14] Fu W., Watanabe Y., Inoue K., Moriguchi N., Fusa K., Yanagisawa Y., Mutoh T., Nakamura T. (2018). Effects of Pre-Cooked Cheeses of Different Emulsifying Conditions on Mechanical Properties and Microstructure of Processed Cheese. Food Chem..

[15] Salek R.N., Vašina M., Lapčík L., Černíková M., Lorencová E., Li P., Buňka F. (2019). Evaluation of Various Emulsifying Salts Addition on Selected Properties of Processed Cheese Sauce with the Use of Mechanical Vibration Damping and Rheological Methods. LWT.

[16] Talbot-Walsh G., Selomulya C. (2021). The Effect of Rennet Casein Hydration on Gel Strength and Matrix Stability of Block-Type Processed Cheese. Food Struct..

[17] Vollmer A.H., Kieferle I., Pusl A., Kulozik U. (2021). Effect of Pentasodium Triphosphate Concentration on Physicochemical Properties, Microstructure, and Formation of Casein Fibrils in Model Processed Cheese. J. Dairy Sci..

[18] Eckner K.F., Dustman W.A., Rys-Rodriguez A.A. (1994). Contribution of Composition, Physicochemical Characteristics and Polyphosphates to the Microbial Safety of Pasteurized Cheese Spreads. J. Food Prot..

[19] Loessner M.J., Maier S.K., Schiwek P., Scherer S. (1997). Long-Chain Polyphosphates Inhibit Growth of *Clostridium tyrobutyricum* in Processed Cheese Spreads. J. Food Prot..

[20] Lee H., Lee S., Kim S., Lee J., Ha J., Yoon Y. (2016). Quantitative Microbial Risk Assessment for *Clostridium perfringens* in Natural and Processed Cheeses. Asian-Australas. J. Anim. Sci..

[21] Oliveira R.B.A., Baptista R.C., Chincha A.A.I.A., Conceição D.A., Nascimento J.S., Costa L.E.O., Cruz A.G., Sant’Ana A.S. (2018). Thermal Inactivation Kinetics of *Paenibacillus sanguinis* 2301083PRC and *Clostridium sporogenes* JCM1416MGA in Full and Low Fat “Requeijão Cremoso”. Food Control.

[22] Oliveira R.B.A., Lopes L.S., Baptista R.C., Chincha A.A.I.A., Portela J.B., Nascimento J.S., Costa L.E.O., Cruz A.G., Sant’Ana A.S. (2018). Occurrence, Populations, Diversity, and Growth Potential of Spore-Forming Bacteria in “Requeijão Cremoso”. LWT Food Sci. Technol..

[23] Fusieger A., da Silva R.R., de Jesus Silva S.R., Honorato J.A., Teixeira C.G., Souza L.V., Magalhães I.N.S., da Silva Costa N.A., Walter A., Nero L.A. (2022). Inhibitory Activity of an Emulsifying Salt Polyphosphate (JOHA HBS^®^) Used in Processed Cheese: An in Vitro Analysis of Its Antibacterial Potential. LWT Food Sci. Technol..

[24] (2004). Microbiology of Food and Animal Feeding Stuffs—Horizontal Method for the Enumeration of Presumptive Bacillus cereus Colony Count Technique at 30 Degrees.

[25] Zhao X., da Silva M.B.R., Van der Linden I., Franco B.D.G.M., Uyttendaele M. (2021). Behavior of the Biological Control Agent *Bacillus thuringiensis* subsp. aizawai ABTS-1857 and Salmonella enterica on Spinach Plants and Cut Leaves. Front. Microbiol..

[26] (2004). Microbiology of Food and Animal Feeding Stuffs—Horizontal Method for the Enumeration of Clostridium perfringens—Colony-Count Technique.

[27] (2003). Microbiology of the Food Chain—Horizontal Method for the Enumeration of Microorganisms—Part 1: Colony Count at 30 °C by the Pour Plate Technique.

[28] Wickham H. (2016). Ggplot2: Elegant Graphics for Data Analysis.

[29] Buňková L., Pleva P., Buňka F., Valášek P., Kráčmar S. (2008). Antibacterial Effects of Commercially Available Phosphates on Selected Microorganisms. Acta Univ. Agric. Silvic. Mendel. Brun..

[30] Lorencová E., Vltavská P., Budinský P., Koutný M. (2012). Antibacterial Effect of Phosphates and Polyphosphates with Different Chain Length. J. Environ. Sci. Health Part A.

[31] Catania A.M., Civera T., di Ciccio P.A., Grassi M.A., Morra P., Dalmasso A. (2021). Characterization of Vegetative *Bacillus cereus* and *Bacillus subtilis* Strains Isolated from Processed Cheese Products in an Italian Dairy Plant. Foods.

[32] Ehling-Schulz M., Lereclus D., Koehler T.M. (2019). The *Bacillus cereus* Group: *Bacillus* Species with Pathogenic Potential. Microbiol. Spectr..

[33] Bağcıoğlu M., Fricker M., Johler S., Ehling-Schulz M. (2019). Detection and Identification of *Bacillus cereus*, *Bacillus cytotoxicus*, *Bacillus thuringiensis*, *Bacillus mycoides* and *Bacillus weihenstephanensis* via Machine Learning Based FTIR Spectroscopy. Front. Microbiol..

[34] Maier S.K., Scherer S., Loessner M.J. (1999). Long-Chain Polyphosphate Causes Cell Lysis and Inhibits *Bacillus cereus* Septum Formation, Which Is Dependent on Divalent Cations. Appl. Environ. Microbiol..

[35] Akhtar S., Paredes-Sabja D., Sarker M.R. (2008). Inhibitory Effects of Polyphosphates on *Clostridium perfringens* Growth, Sporulation and Spore Outgrowth. Food Microbiol..

[36] Christ J.J., Blank L.M. (2018). Enzymatic Quantification and Length Determination of Polyphosphate down to a Chain Length of Two. Anal. Biochem..

[37] Lee R.M., Hartman P.A., Stahr2 H.M., Olson3 D.G., Williams F.D. (1994). Antibacterial Mechanism of Long-Chain Polyphosphates in *Staphylococcus aureus*. J. Food Prot..

[38] Buňka F., Doudová L., Weiserová E., Černíková M., Kuchař D., Slavíková Š., Nagyová G., Ponížil P., Grůber T., Michálek J. (2014). The Effect of Concentration and Composition of Ternary Emulsifying Salts on the Textural Properties of Processed Cheese Spreads. LWT Food Sci. Technol..

[39] Nagyová G., Buňka F., Salek R.N., Černíková M., Mančík P., Grůber T., Kuchař D. (2014). Use of Sodium Polyphosphates with Different Linear Lengths in the Production of Spreadable Processed Cheese. J. Dairy Sci..

[40] Awad R.A., Abdel-Hamid L.B., El-Shabrawy S.A., Singh R.K. (2004). Physical and Sensory Properties of Block Processed Cheese with Formulated Emulsifying Salt Mixtures. Int. J. Food Prop..

[41] Kim N.H., Lee N.Y., Kim M.G., Kim H.W., Cho T.J., Joo I.S., Heo E.J., Rhee M.S. (2018). Microbiological Criteria and Ecology of Commercially Available Processed Cheeses According to the Product Specification and Physicochemical Characteristics. Food Res. Int..

